# Supplementary material for: Inhibition–Disruption of Candida glabrata Biofilms: Symmetrical Selenoesters as Potential Anti-Biofilm Agents
Source: Microorganisms. 2019 Dec 9;7(12):664. doi: 10.3390/microorganisms7120664 (PMC6955995; doi:10.3390/microorganisms7120664)
Supplement: Supplementary file 1 [file microorganisms-07-00664-s001.pdf]

Supplementary Materials

Inhibition–Disruption of *Candida Glabrata* Biofilms: Symmetrical Selenoesters as Potential Anti-Biofilm Agents

María L. De la Cruz-Claure [1,*], Ariel A. Cèspedes-Llave [2], María T. Ulloa [3], Miguel Benito-Lama [4], Enrique Domínguez-Álvarez [4] and Agatha Bastida [4,*]

[1] Facultad de Odontología. Facultad de Ciencias de Bioquímica, Química Farmacéutica y Biología. Universidad Mayor, Real y Pontificia de San Francisco Xavier de Chuquisaca. Sucre, Bolivia Calle Dalence Casilla 497, Sucre, Bolivia; mariafeliz3@hotmail.com

[2] Facultad de Ciencias de Bioquímica, Química Farmacéutica y Biología. Universidad Mayor, Real y Pontificia de San Francisco Xavier de Chuquisaca. Sucre, Bolivia Calle Dalence Casilla 497, Sucre, Bolivia; cespedes.ariel@usfx.bo

[3] Programa de Microbiología-Micología, Facultad de Medicina. Universidad de Chile (Av. Independencia 1027, Comuna Independencia) Santiago, Chile; mtulloa@med.uchile.cl

[4] Consejo Superior de Investigaciones Científicas, Instituto de Química Orgánica General, (IQOG-CSIC) c/Juan de la Cierva 3, 28006-Madrid, Spain; miguelbenitodelama@gmail.com (M.B.-L.); e.dominguez-alvarez@iqog.csic.es (E.D.-A.)

* Correspondence: agatha.bastida@csic.es (A.B.); Tel.: +34-5618806 (A.B.)

Table S1. Biofilm formation of 11 *C. glabrata* dental isolates and *C. glabrata* ATCC2001 control strain.

Candida specie	Biofim formation on PVC
C. glabrata ATCC 2001	+
**Isolate denture Strain number**	
S1	+
S2	+
S3	+
S5	+
S10	+
S15	+
S16	+
S18	+
S22	+
S47	+
S49	+

Table S2. MIC values of voriconazole (VOR), caspofungin (CAS), micafungin (MIC), amphotericin B(APH) and fluconazole(FLZ) against *C. glabrata* (*N* = 37 dental isolates).

	MIC values (ug/ml)				
**Strain number**	**VOR**	**CAS**	**MCA**	**AMB**	**FLZ**
1	0,25	0,25	≤<=0,06	0,5	0.094
2	0,25	0,25	≤<=0,06	0,5	0.094
3	0,25	0,25	≤<=0,06	0,5	0,094
4	0,25	0,25	≤<=0,06	0,5	0,125
5	O,5	>=8	4	1	0,064
6	0,25	0,25	≤<=0,06	0,5	0,125
7	0,25	0,25	≤<=0,06	0,5	0,094

8	0,25	0,25	≤<=0,06	0,5	0,094
9	0,25	0,25	≤<=0,06	0,5	0,094
10	0,25	0,25	≤<=0,06	0,5	0,125
11	0,25	0,25	≤<=0,06	0,5	0,064
12	≤0,12	1	≤<=0,06	0,5	8
13	≤0,12	≤8	4	0,5	≤32
14	0,25	0,25	≤0,06	0,5	0,125~~≤<=1~~
15	0,25	0,25	≤0,06	1	≤2
16	0,25	0,25	≤0,06	0,5	0,094
17	0,25	0,25	≤0,06	1	0,064
18	≤0,12	0,25	≤0,06	0,5	0,19
20	≤0,12	0,25	≤0,06	0,5	0.08
21	0,25	0,25	≤0,06	0,5	4
22	≤0,12	0,25	≤0,06	0,5	4
23	≤0,12	0,25	≤0,06	0,5	2
24	<=0,12	0,25	≤0,06	0,5	4
25	≤0,12	0,25	≤0,06	1	0,094
29	0,25	≥8	≤0,06	0,5	0,125
30	0,25	0,25	≤0,06	0,5	0,125
31	≤12	≥8	4	0,5	32
32	0,5	0,25	≤6	0,5	0,049
33	1	≤0,12	≤0,05	1	0,19
34	0,25	0,5	≤0,06	0,5	0,094
35	≤0,12	≤0,12	≤0,06	1	0,064
36	0,25	1	≤0,06	0,5	0,19
37	0,25	0,5	≤0,06	0,5	8
38	0,25	≤0,12	≤0,07	0,5	32
39	0,25	0,25	≤0,06	0,5	0,19
47	0,25	0,25	≤0.06	0,5	≥32
49	≤0,12	0,25	≤0,06	0,5	0,19<br>≤1

Table S3.- Biofilm inhibition IC$_{50}$ and dispersion EC$_{50}$ of fluoride and seleno-esters compounds against *C. glabrata*.

**Compounds**	**IC$_{50}$ (g/ml)***	**EC$_{50}$ (g/ml)****
**F-1**	50	>150
**F-2**	100	>300
**F-3**	150	>320
**Se-5**	2	2-4
**Se-7**	1-2	>4
**Se-8**	10	>4
Caspofungin	1	>6

*: IC$_{50}$, the concentration of the compounds that inhibits 50% biofilm development

****:** EC$_{50}$, the concentration of the fluoride, selenoester compounds 50% biofilm formed by *C. glabrata*.

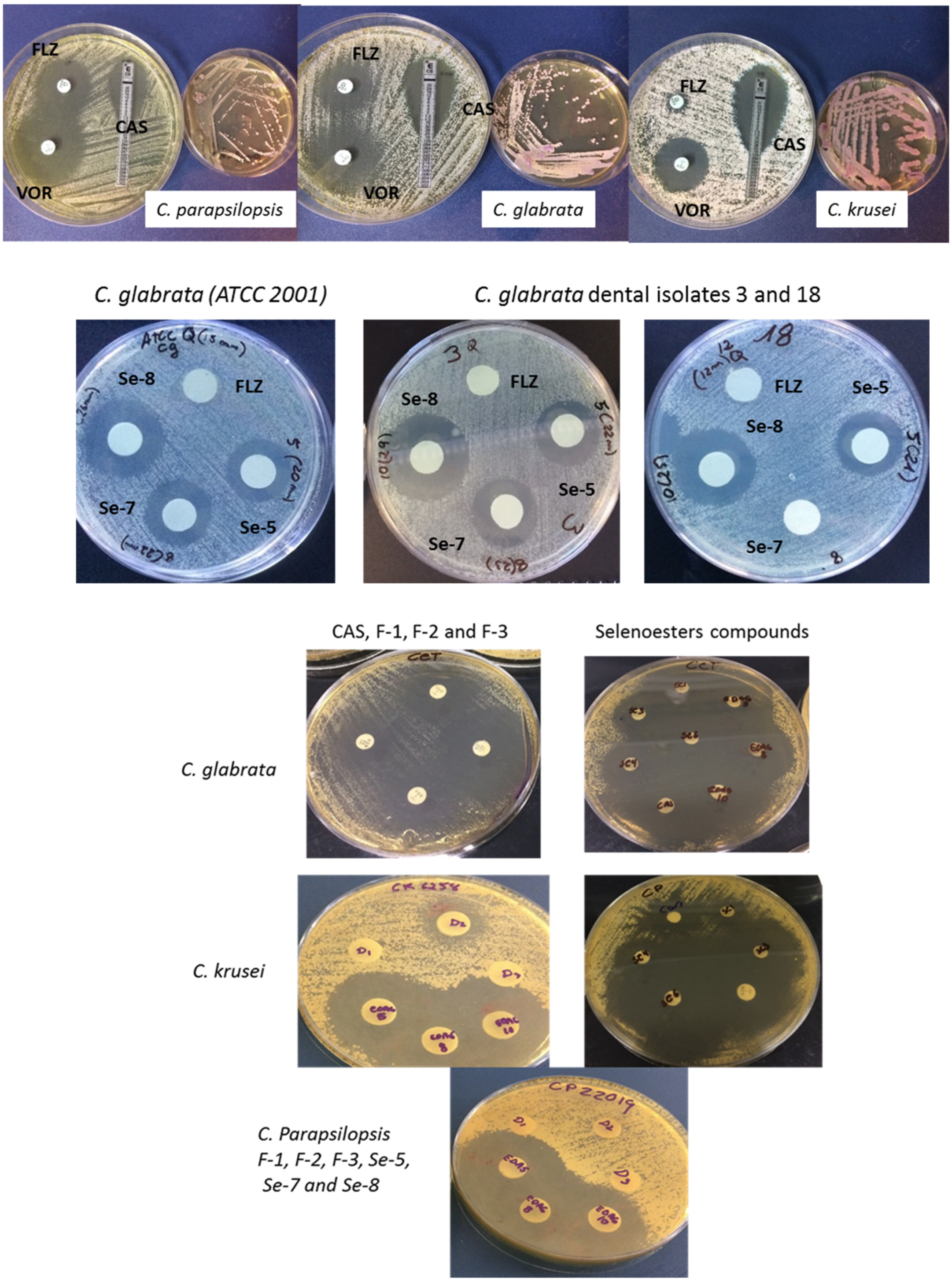

Figure S1. Zone diameter wholes (mm) for casponfunginvoriconazole, fluconazole, fluoride and selenoester compounds against *Candida* spp. according to the agar diffusion method and E test.

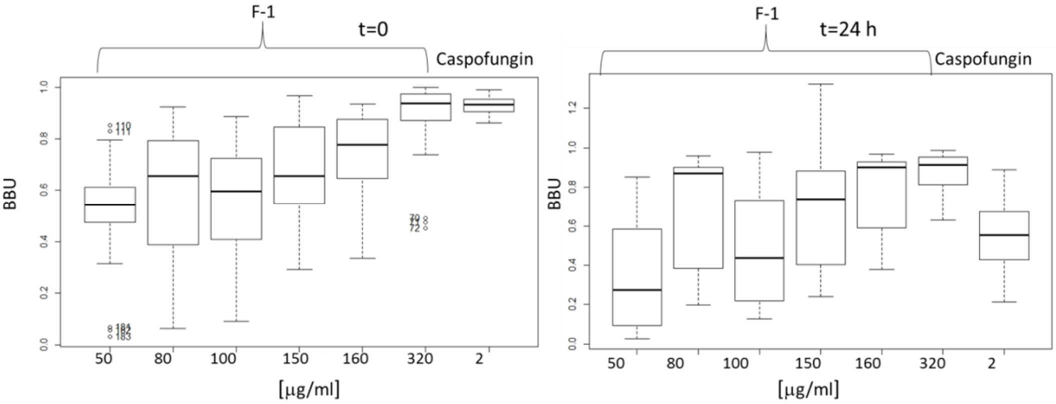

Figure S 2.- Mean values of BBU for *C. glabrata* ATCC 2001 with **F-1** (50–320 g/ml) at t = 0 and 24 h. As control was used caspofungin (2 g/ml). Error bars indicate the SDs. BBU = 1 it correspond to 100% inhibition of biofilm formation and BBU = 0 do not affect in the biofilm formation/disruption.

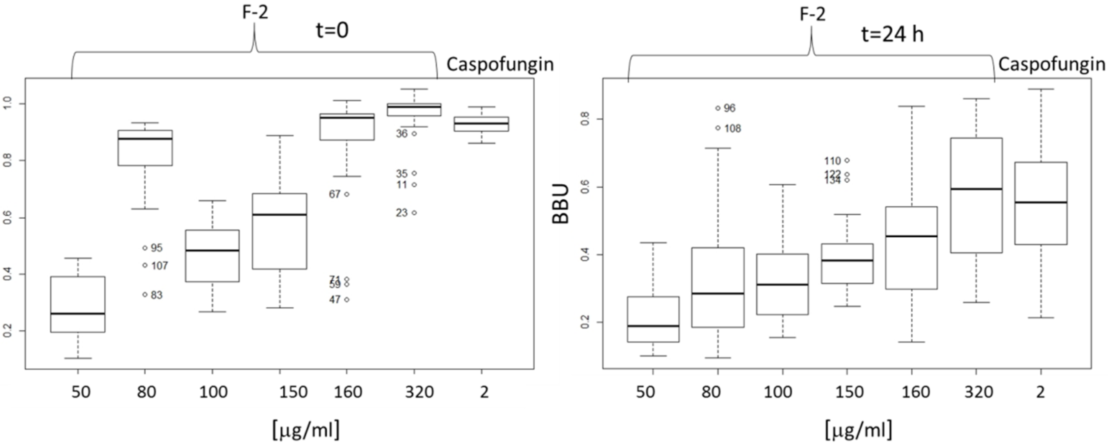

Figure S3.- Mean values of BBU for *C. glabrata* ATCC 2001 with **F-2** (50–320 g/ml) at t = 0 and 24 h. As control was used caspofungin (2 g/ml). Error bars indicate the SDs. BBU = 1 it correspond to 100% inhibition of biofilm formation (t = 24 h) or destroy (t = 0 h) ≥and BBU=0 do not affect in the biofilm formation/destroy.

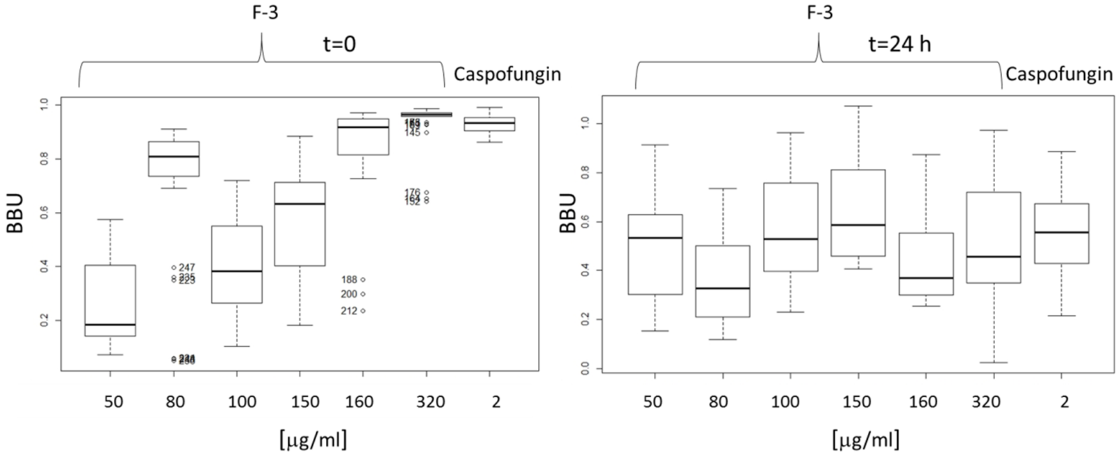

Figure S4.- Mean values of BBU for *C. glabrata* ATCC 2001 with **F-3** 50-320 g/ml) at t = 0 and 24 h. As control was used caspofungin (2 g/ml). Error bars indicate the SDs. BBU = 1 it correspond

to 100% inhibition of biofilm formation (t = 24 h) or destroy (t = 0 h) and BBU = 0 do not affect in the biofilm formation/destroy.

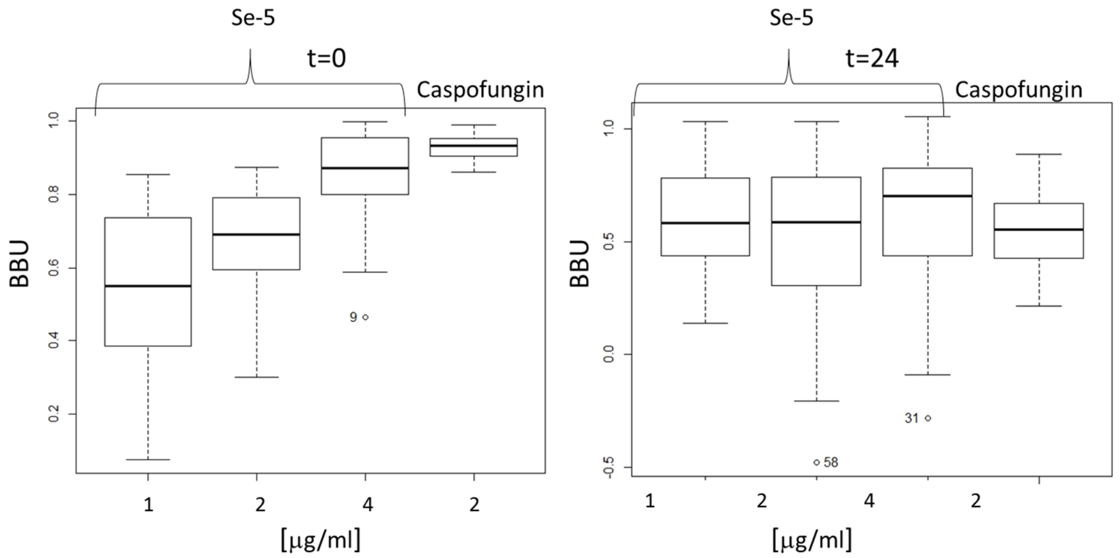

Figure S 5.- Mean values of BBU for *C. glabrata* ATCC 2001 with **Se-5** (50–320   g/ml) at t = 0 and 24 h. As control was used caspofungin (2 g/ml). Error bars indicate the SDs. BBU = 1 it correspond to 100% inhibition of biofilm formation (t = 24 h) or destroy (t = 0 h) and BBU = 0 do not affect in the biofilm formation/dispersion.

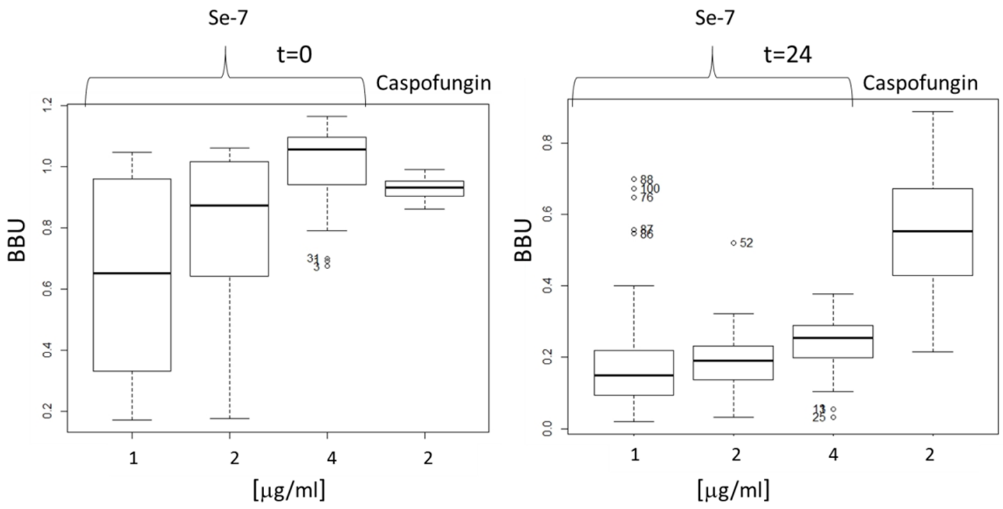

Figure 6.- Mean values of BBU for *C. glabrata* ATCC 2001 with **Se-7** (50–320   g/ml) at t = 0 and 24 h. As control was used caspofungin (2 g/ml). Error bars indicate the SDs. BBU = 1 it correspond to 100% inhibition of biofilm formation (t = 24 h) or destroy (t = 0 h) and BBU = 0 do not affect in the biofilm formation/dispersion.

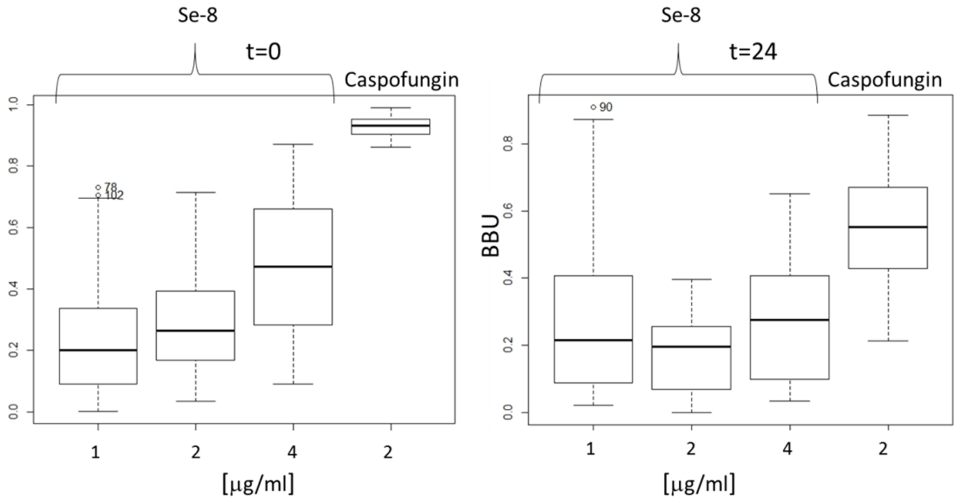

Figure S7.- Mean values of BBU for *C. glabrata* ATCC 2001 with **Se-8** (50–320 g/ml) at t = 0 and 24 h. As control was used caspofungin (2 g/ml). Error bars indicate the SDs. BBU = 1 it correspond to 100% inhibition of biofilm formation (t = 24 h) or destroy (t = 0 h) and BBU = 0 do not affect in the biofilm formation/dispersion.
